# Age of introduction of first complementary feeding for infants: a systematic review

**DOI:** 10.1186/s12887-015-0409-5

**Published:** 2015-09-02

**Authors:** Wafaa Qasem, Tanis Fenton, James Friel

**Affiliations:** Department of Human Nutritional Sciences, University of Manitoba, Winnipeg, MB R3T 2N2 Canada; Richardson Centre for Functional Foods and Nutraceuticals, 196 Innovation Drive, University of Manitoba, Winnipeg, MB R3T 6C5 Canada; Nutrition Services, Alberta Health Services, Alberta Children’s Hospital Research, Institute, Department of Community Health Sciences, University of Calgary, TRW Building, 3280 Hospital Dr NW, Calgary, AB T2N 4Z6 Canada

**Keywords:** Age of introduction of solids, Breastfed infant, Complementary feeding, Growth, Iron, Solid food

## Abstract

**Background:**

Despite a World Health Organization recommendation for exclusive breastfeeding of all full-term infants to 6 months of age, it is not clear what the health implications may be. Breast milk alone may not meet the nutrition needs for all growing infants, leaving them at risk for deficiencies. The objective of this study was to investigate the relationship between moderate (4 months) versus late (6 months) introduction of complementary foods to the full-term breastfed infant on iron status and growth.

**Methods:**

An electronic search of peer-reviewed and gray-literature was conducted for randomized control trials (RCTs) and observational studies related to the timing of introduction of complementary foods. Iron status and growth data from the relevant RCTs were analyzed using RevMan 5.2.11.

**Results:**

Three RCTs and one observational study met the inclusion criteria. Meta-analysis showed significantly higher hemoglobin levels in infants fed solids at 4 months versus those fed solids at 6 months in developing countries [mean difference [MD]: 5.0 g/L; 95 % CI: 1.5, 8.5 g/L; *P* = 0.005]. Meta-anaysis also showed higher serum ferritin levels in the 4-month group in both developed and developing countries [MD: 26.0 μg/L; 95 % CI: -0.1, 52.1 μg/L, *P* = 0.050], [MD: 18.9 μg/L; 95 % CI: 0.7, 37.1 μg/L, *P* = 0.040]. Short follow-up periods and small sample sizes of the included studies were the major limitations.

**Conclusions:**

RCT evidence suggests the rate of iron deficiency anemia in breastfed infants could be positively altered by introduction of solids at 4 months.

**Electronic supplementary material:**

The online version of this article (doi:10.1186/s12887-015-0409-5) contains supplementary material, which is available to authorized users.

## Background

The World Health Organization (WHO) currently recommends exclusively breastfeeding infants for the first 6 months of life, followed by introduction of adequate complementary foods (CF). This recommendation is for infants living in developing and developed countries, including Canada [1, 2]. Although there is nearly universal agreement that breast milk alone is the optimal first food, the age range in which solids should be introduced is less clear, leading to “weanling’s dilemma” [3].

The complementary feeding period accompanies a critical window of vulnerability. During this time period, failure to grow is a significant concern [4]. Micronutrient deficiencies can also occur during this period, mostly because infants have higher nutrient demands relative to increased energy requirements. Deficiencies of certain micronutrients such as iron result in potentially irreversible negative effects on brain development and other detrimental psychological outcomes [5]. There is general, but not universal, agreement that the iron stores of infants start to deplete at about 6 months of age, leaving the infants at high risk of iron deficiency and iron deficiency anemia. This is especially true among exclusively breastfed infants [6, 7]. The estimated prevalence of iron deficiency anemia among Canadian children aged 1–5 years is 5 % and was found to be five times higher among Inuit children [8, 9]. Therefore, it is important to determine the ideal age to introduce iron-rich CF. Our objectives were to evaluate the current scientific evidence and to investigate the relationship between time of introduction of CF with iron status and growth in breastfed infants. This review includes any relevant studies that targeted exclusively breastfed infants between 4 and 6 months of age.

## Methods

Our review was conducted according to the PRISMA guidelines [10]. The Cochrane Risk of Bias Tool [11] was used to assess study quality by the two reviewers. Any disagreements were resolved through discussion.

### Literature search

Electronic searches of the MEDLINE and CINHAL databases were used to identify publications regarding the timing of introduction of CF. The searches were completed by two authors (WQ, TRF) in May, 2014. Medical subject headings and text keywords used to search included: complementary feeding, infant food, solid(s), weaning, timing of introduction, micronutrient, iron, developmental outcomes, iron supplementation, random allocation, cohort studies, follow up studies, prospective studies, cross over studies, and cross sectional studies. To decrease the chance of publication bias influencing the results, TRF conducted a gray literature search to include studies that may not be included in bibliographic retrieval systems. Google, Current Controlled Trials, NIH Clinical Research Trials, ISRCTN, and Cochrane Register of Clinical Trials were also searched up to May, 2014.

### Inclusion criteria

We included any randomized controlled trials (RCTs) and observational studies that focused on introduction of CF at 4 months versus 6 months of age. All included studies were conducted on healthy, full-term, exclusively breastfed infants.

### Exclusion criteria

Studies were excluded if they included formula-fed, preterm, or low birth weight infants or involved medicinal iron supplementation. Studies in which infants were introduced to solid foods at ages younger than 4 months or greater than 6 months of age were also excluded.

### Data analysis

Meta-analyses were performed on all of the iron and growth data from included RCTs, regardless of the number of RCTs, following Kramer and Kakuma’s systematic review approach [12]. Weighted mean difference meta-analysis was carried out using Review Manager software (RevMan Version 5.2.11, The Cochrane Collaboration, London, UK) [13] to assess the effect of age of introduction of solids on iron status and linear growth (weight, length and head circumference). The analyses were stratified by developing versus developed country and by study design (e.g., randomized controlled trials versus observational studies).

## Results

**Fig. 1:**
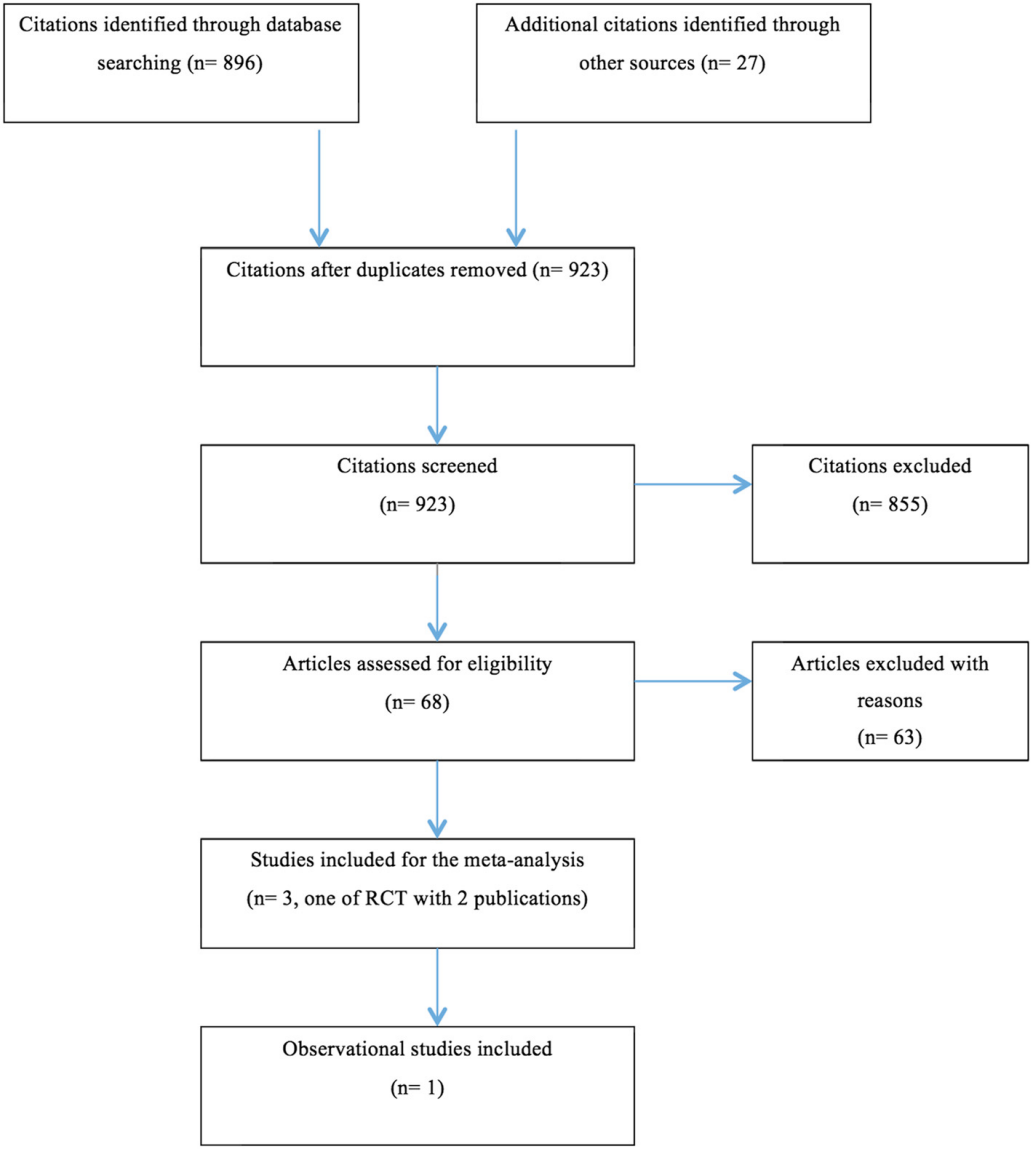
Study flow of the systematic review

A total of 923 study citations were found related to age of complementary feeding (Fig. 1). Twenty-five RCTs were found, only three of which met the inclusion criteria. One was conducted in a developed country (generating two separate publications), and two were in developing countries (Table 1). Forty-seven observational studies examining the age of introduction of CF were located. Only one of the observational studies (in a developing country) met the inclusion criteria (Table 1). Table 2 lists the excluded studies and the reasons for their exclusion.

**Table 1:** Summary of results of studies included in the systematic review

Study	Study design	Country	N	Outcomes related to age of CF introduction	Results from CF introduction at			*P*	Conclusion/Main findings related to age of introduction of solids
						6 mo	4 mo		
Cohen et al. 1994 [14]	RCT	Honduras	141	Growth	Wt gain (g)	1092 (356)	1051 (315)	˃0.05	No sig differences in weight and length gain were found between the groups.
					Length gain (cm)	3.9 (1.2)	3.8 (1.1)	˃0.05	
Dewey et al. 1998 [18]	RCT	Honduras	164	Fe status	Hb (g/L)	104 (10)	109 (10)	˂0.05	Infants who received CF at 4 months had sig higher iron status parameters than EBF infants
					Ht	0.33 (0.027)	0.34 (0.026)	<0.05	
					Ferritin (μ/L)	48.4 (44.2)	67.3 (64.5)	˂0.05	
Jonsdottir et al. 2012 [15]	RCT	Iceland	100	Growth	Wt gain (z score)	-0.01(0.42)	-0.02(0.31)	0.90	No sig differences were found between the groups in growth. Sig positive effect of earlier CF introduction on iron stores
					Length gain (z score)	0.04 (0.51)	0.03 (0.50)	0.96	
					Gain in HC (z score)	0.06 (0.48)	0.06 (0.40)	0.99	
				Fe status	Hb (g/L)	113.7 (7.3)	113.9 (6.1)	0.91	
					Ferritin (μg/L)	44.0 (53.8)	70.0 (77.3)	0.02	
Wells et al. 2012 [16]	RCT	Iceland	100	Growth	Wt (z score)	0.36 (0.99)	0.28 (1.08)	0.7	No significant differences were found between the groups in growth and body composition.
					Length (z score)	0.77 (0.84)	0.60 (0.92)	0.3	
					BMI (z score)	-0.10 (1.04)	-0.08 (1.14)	0.9	
					HC (z score)	1.02 (0.89)	0.94 (0.77)	0.6	
				Body composition	Lean mass (kg)	4.96 (1.18)	5.13 (0.92)	0.4	
					Fat mass (kg)	3.04 (1.12)	2.71 (0.96)	0.14	
Khadivzadeh and Parsai 2004 [17]	Observ.	Islamic republic of Iran	200	Growth	Wt (g)	7719 (763)	7762 (843)	0.95	There were no significant differences in wt and length between infants fed solids at 4 months and infants fed solids at 6 mo of age.
					Length (cm)	66.5 (3.0)	66.6 (3.1)	0.86	
					Wt gain (g)	922 (500)	1015 (419)	0.86	
					Length gain (cm)	3.6 (1.3)	3.5 (1.1)	0.70	

N.B: *BMI* body mass index, *CF* complementary feeding, *EBF* exclusively breastfeeding, *HC* head circumference, *Ht* hematocrit, *mo* month, *Observ*. observational, *Wt* weight. Data are presented as mean (SD). Jonsdottir et al. 2012 [15] and Wells et al. 2012 [16] were two articles published from a single RCT

**Table 2:** Excluded studies

Study (design)	Reason behind exclusion
Adu-Afarwuah et al. 2007 [28] (RCT)	Age of introduction of solids > 6 mo
Bisimwa et al. 2012 [29] (RCT)	Age of introduction of solids > 6 mo
Fewtrell et al. 2012 [30] (RCT)	Age of introduction of solids > 6 mo
Gibson et al. 2011 [31] (RCT)	Age of introduction of solids > 6 mo
Hambidge et al. 2004 [32] (RCT)	Age of introduction of solids > 6 mo
Krebs et al. 2011 [33] (RCT)	Age of introduction of solids > 6 mo
Ly et al. 2006 [34] (RCT)	No EBF group (no control group)
Martin-Calama et al. 1997 [35] (RCT)	Age of introduction of solids < 4 mo
Mehta et al. 1998 [36] (RCT)	Age of introduction of solids < 4 mo
Mosley et al. 2001 [37] (RCT)	Preterm infants
Nicoll et al. 1982 [38] (RCT)	Newborn infants
Ojofeitimi and Elegbe 1982 [39] (RCT)	Newborn infants
Phuka et al. 2008 [40] (RCT)	Age of introduction of solids > 6 mo
Rivera et al. 2004 [41] (RCT)	Age of introduction of solids non specified
Roy 2006 [42] (RCT)	Age of introduction of solids > 6 mo. Malnourished infants
Sachdev et al. 1991 [43] (RCT)	Water supplementation. Infants age <4 mo
Saleem 2010 [44] (RCT)	Age of introduction of solids > 6 mo
Sarker 2009 [45] (RCT)	Age of introduction of solids > 6 mo. No EBF group
Schutzman et al. 1986 [46] (RCT)	Newborn infants
Simondon et al. 1996 [47] (RCT)	No EBF group
Ziegler et al. 2009 [48] (RCT)	Non EBF
Ahmed et al. 1993 [49]	Age of introduction of solids < 4 mo
Armar-Klemesu et al. 1991 [50]	Age of introduction of solids non specified
Arvas et al. 2000 [51]	Medicinal iron supplementation
Baker et al. 2004 [52]	Age of introduction of solids < 4 mo
Baird et al. 2008 [53]	Mixed feeding (formula + BM)
Calvo et al. 1992 [54]	Age of introduction of solids was at 6 mo for both groups
Castro et al. 2009 [55]	Mixed feeding (formula + BM), no data on postnatal birth wt and conditions
Chantry et al. 2007 [56]	Non EBF (other foods introduced)
Domellöf et al. 2001 [57]	Age of introduction of solids > 6 mo, medicinal iron supplementation
Dube et al. 2010 [58]	No analysis on early vs late introduction of solids among the groups
Durá Travé & Diaz Velaz 2002 [59]	Early weaned group had mixed feeding (formula + BM)
Eissa et al. 1990 [60]	Age of introduction of solids non specified
Filipiak et al. 2007 [61]	Mixed feeding (formula + BM), no EBF group
Forsyth et al. 1993 [62]	Age of introduction of solids < 4 mo
Freeman et al. 1998 [63]	Mixed feeding (formula + BM)
Gray 1996 [64]	Mixed feeding (formula + BM)
Haschke & van’t Hof 2000 [65]	Age of introduction of solids < 4 mo
Heinig et al. 1993 [66]	Mixed feeding (formula + BM), age of introduction of solids = or > 6 months
Hokama 1993 [67]	No analysis on association between age of introduction of solids and iron parameters
Kajosaari & Saarinen 1983 [68]	Age of introduction of solids < 4 mo
Kajosaari 1991 [69]	Age of introduction of solids < 4 mo
Kikafunda et al. 2009 [70]	Age of introduction of solids > 6 mo
Kramer et al. 2011 [71]	Age of introduction of solids at 1, 2, 3 mo
Lartey et al. 1999 [72]	Age of introduction of solids > 6 mo
López-Alarcón et al.1997 [73]	Age of introduction of solids < 4 mo
Marlin et al. 1980 [74]	Age of introduction of solids < 4 mo
Marquis et al. 1997 [75]	Infants age group 12-15 mo
Messiah et al. 2012 [76]	Non specific information on how exclusive breastfeeding in BF and in CF groups
Nielsen et al.1998 [77]	No analysis on association between age of introduction of solids among EBF and growth
Piwoz et al. 1996 [78]	Age of introduction of solids < 4 mo
Popkin et al. 1990 [79]	Age of introduction of solids non specified
Quigley et al. 2009 [80]	No analysis on the type of milk received by CF group
Rowland et al. 1988 [81]	Age of introduction of solids non specified
Saarinen & Siimes 1978 [82]	Age of introduction of solids < 4 mo. Mixed feeding (formula + BM)
Salmenpera et al. 1985 [83]	Age of introduction of solids < 4 mo
Simondon & Simondon 1997 [84]	Age of introduction of solids < 4 mo
Sloan et al. 2008 [85]	Age of introduction of solids < 4 mo
Victora et al. 1998 [86]	Age of introduction of solids < 4 mo, low birth weight infants included in the analysis
Wilson et al. 1998 [87]	Age of introduction of solids < 4 mo
Wilson et al. 2006 [88]	Age of introduction of solids < 4 mo
Zhou et al. 2012 [89]	Age of introduction of solids > 6 mo

N.B: *CF* complementary feeding, *EBF* exclusively breastfeeding, *mo* month

### Iron

A total of two RCTs assessed iron status outcomes (Table 1). Meta-analysis (Fig. 2.1) suggested that introduction of solids at 4 months of age did not improve hemoglobin status of breastfed infants in developed countries compared with introduction at 6 months of age [mean difference [MD]: 0.2 g/L; 95 % CI: -2.4, 2.8 g/L; *P* = 0.88]. In developing countries, however (Fig. 3.1), significant improvement was detected with the earlier introduction of solids [MD: 5.0 g/L; 95 % CI: 1.5, 8.5 g/L; *P* = 0.005]. Plasma ferritin concentration was improved with introduction of solids at 4 months of age for infants living in both developed and developing countries [MD: 26.0 μg/L; 95 % CI: −0.1, 52.1 μg/L, *P* = 0.050], [MD: 18.9 μg/L; 95 % CI: 0.7, 37.1 μg/L, *P* = 0.040] (Figs. 2.2 & 3.2). The included observational study did not include iron parameters.

**Fig. 2:**
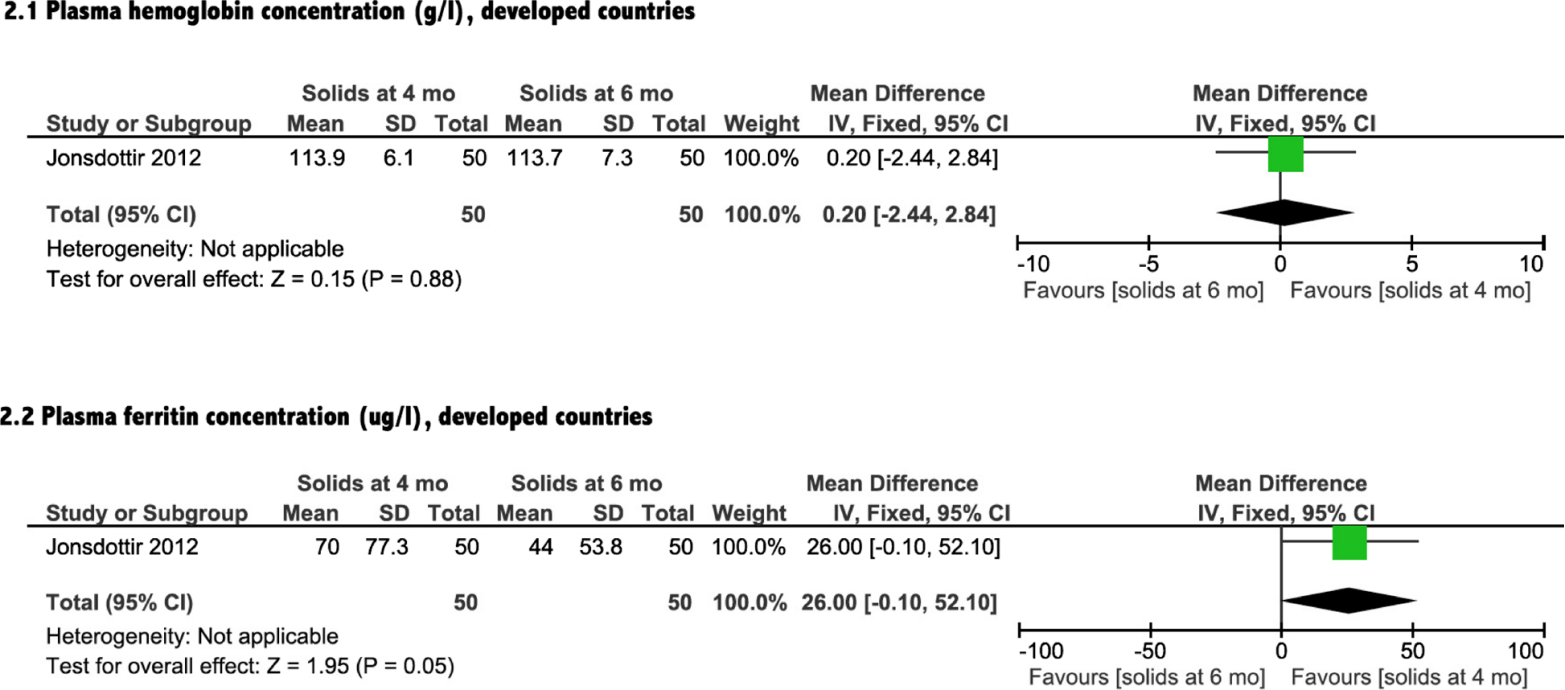
Iron status analysis from developed countries

**Fig. 3:**
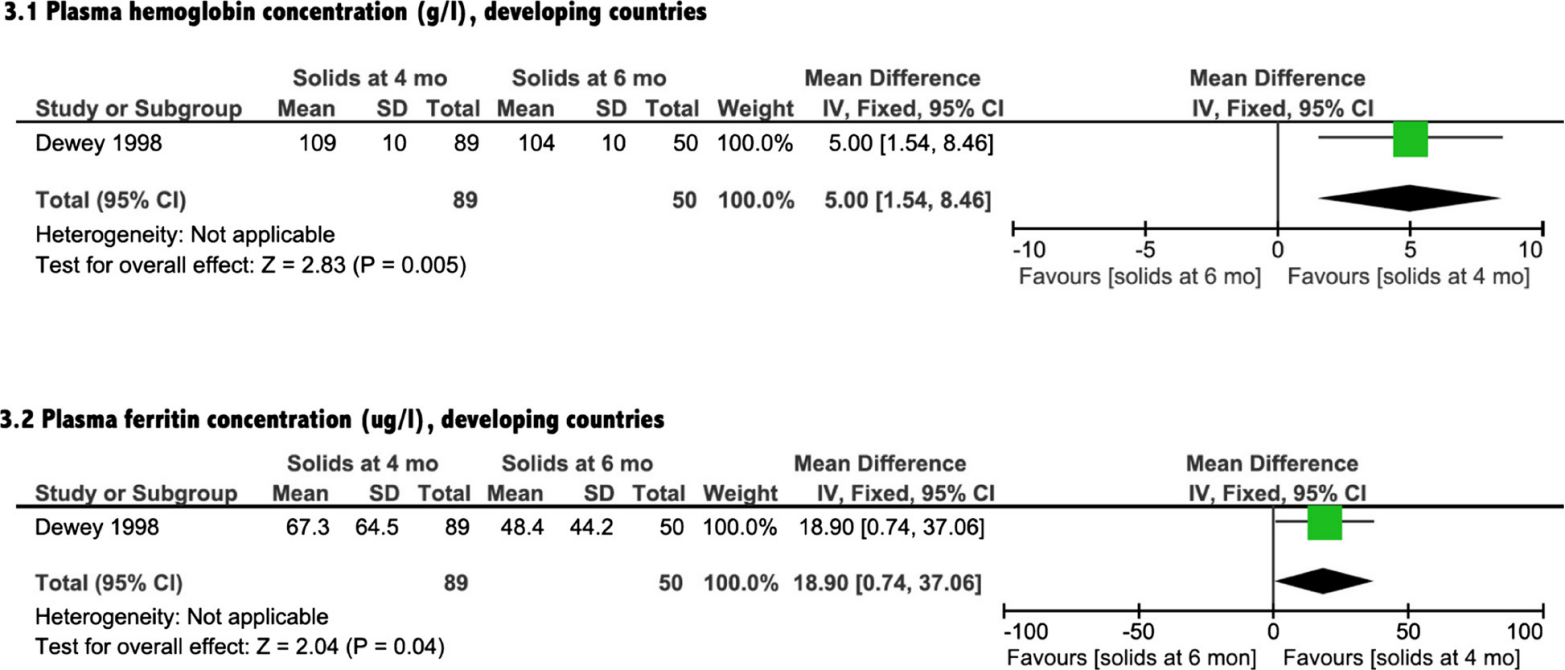
Iron status analysis from developing countries

### Growth

**Fig. 4:**
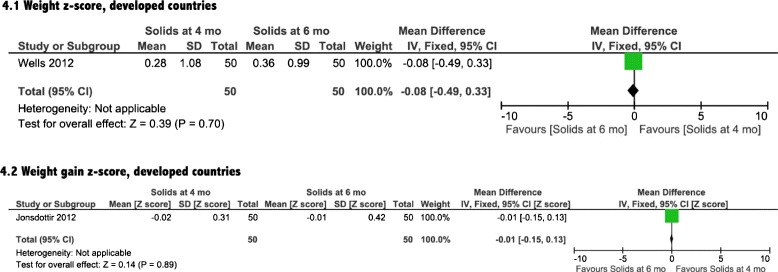
Weight analysis from developed countries

**Fig. 5:**
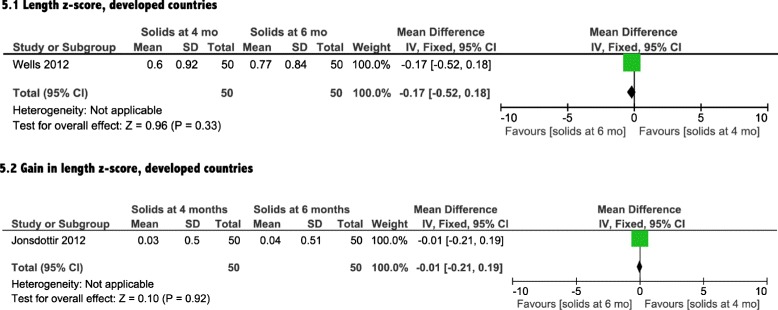
Length analysis from developed countries

**Fig. 6:**
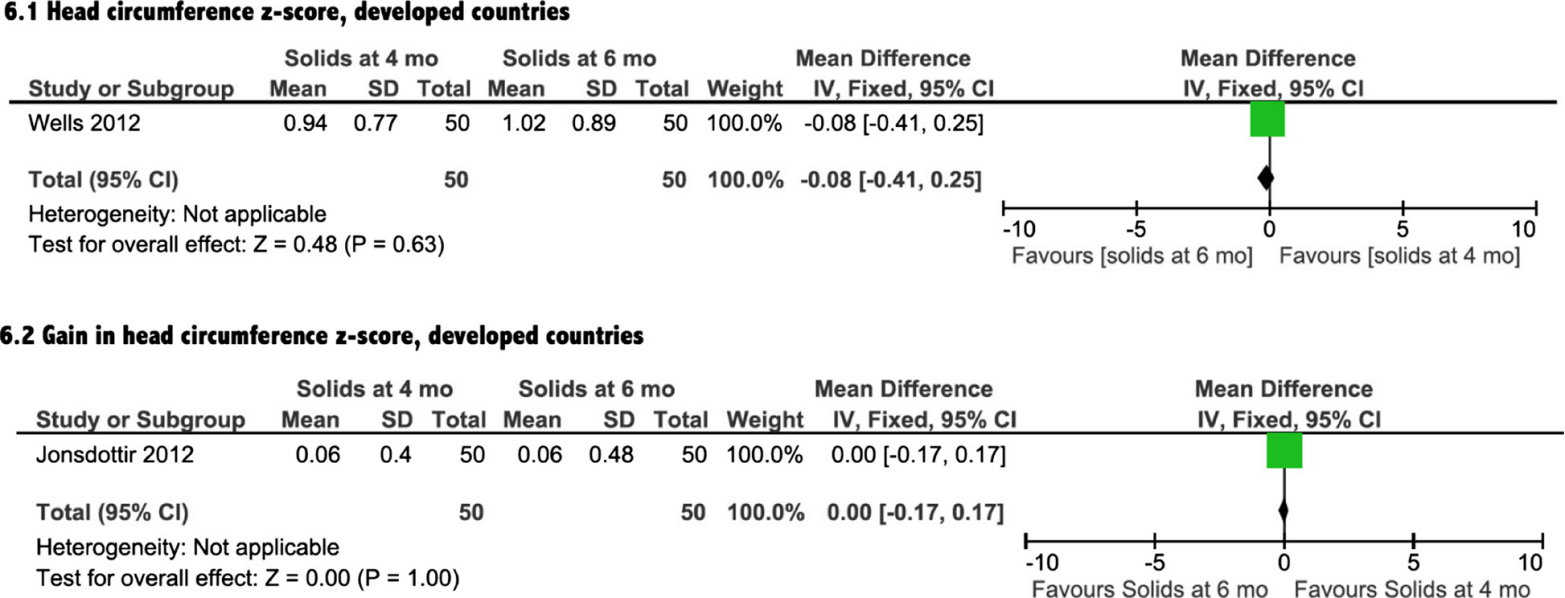
Head circumference analysis from developed countries

**Fig. 7:**
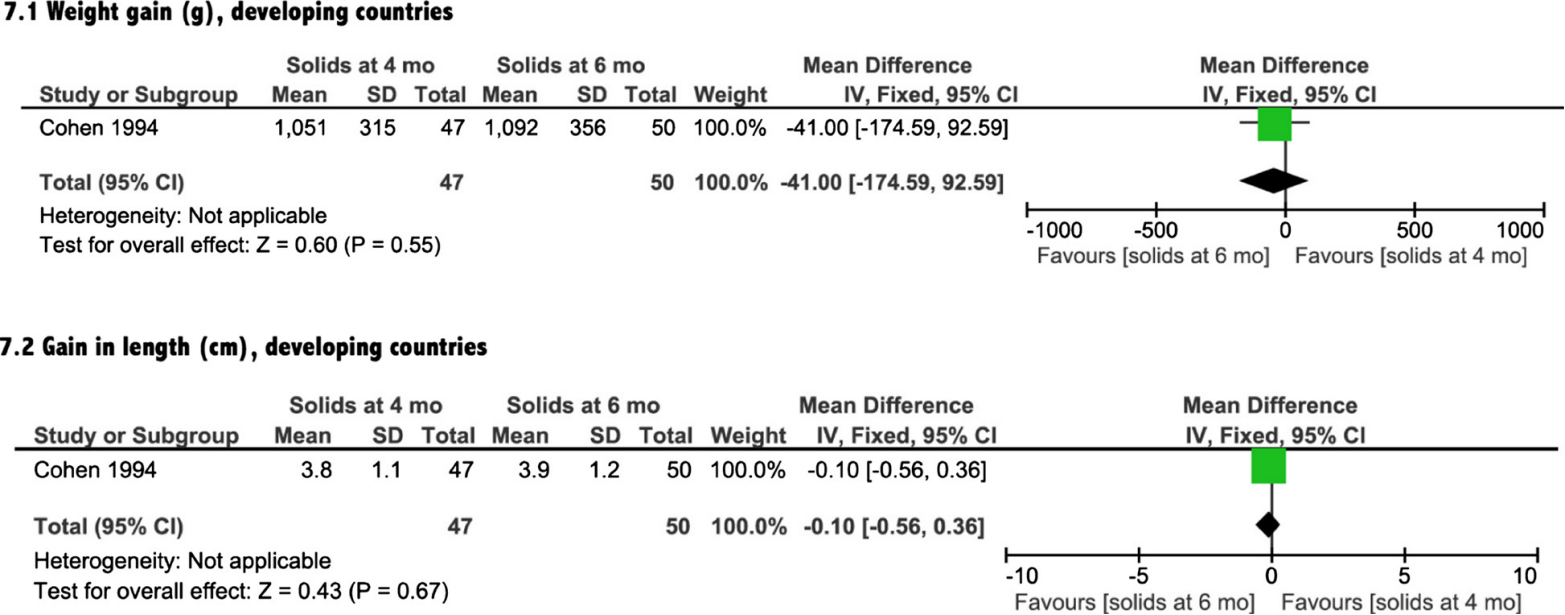
Growth analysis from developing countries

Growth was assessed by differences in weight, length and head circumference. Three [14–16] of the included four interventional studies reported on the impact of introduction of solids on growth (Table 1). The meta-analyses showed a non-significant effect of earlier CF introduction on growth in both developing and developed countries on weight, length and head circumference (Figs. 4, 5, 6, and 7). In addition, the study by Wells et al. (Table 1) showed non-significant differences between the two groups in body composition (lean mass, *P* = 0.4, fat mass, *P* = 0.14).

There was no association between early introduction of complementary foods and a difference in weight and/or length in the study conducted in a developing country (*P* = 0.95, *P* = 0.86, respectively) [17].

### Risk of bias within studies

We assessed the included trials for risk of bias as described in the method section (Table 3). The older studies had moderate risk of bias due to lack of reporting for sequence generation, concealment allocation, and blinding [14, 18]. The two more recent trials [15, 16] had no apparent risk of bias.

**Table 3:** Cochrane Risk of Bias Tool-assessment of studies included in systematic review: individual FODMAPs supplementation

Study	Criteria					
	Adequate sequence generation	Allocation concealment	Blinding	Incomplete outcome data	Free of selective reporting	Free of other bias
Cohen 1994 [14]	Risk	Risk	Risk	Low risk	Low risk	Low risk
Dewey 1998 [18]	Risk	Risk	Risk	Low risk	Low risk	Low risk
Jonsdottir 2012 [15]	Low risk	Low risk	Unclear	Low risk	Low risk	Low risk
Wells 2012 [16]	Low risk	Low risk	Low risk	Low risk	Low risk	Low risk

## Discussion

In this meta-analysis, we found that infants in developing countries who were introduced to solid foods at 4 months of age had clinically relevant increases in hemoglobin and ferritin levels, compared with exclusively breastfed infants at 6 months of age. The data from developed countries showed only a significant increase in ferritin levels in the infants exposed to CF earlier. Our meta-analysis indicated that there was no significant impact of earlier introduction of solids on growth for either developed or developing countries, as evident by a lack of significant differences in weight, length or head circumference measures.

To our knowledge, this is the first systematic review to evaluate the effects of complementary food introduction at 4 versus 6 months of age on iron status and growth. Other reviews have examined the effect of iron-fortified food on iron status and anemia rates on children of different ages [19]. Dewey and Adu-Afaruah reviewed existing studies that looked at the effects of CF on various biochemical and functional outcomes, but they did not evaluate solids introduction at 4 versus 6 months [20]. Systematic reviews/meta-analyses assessing the effect of iron supplementation/fortification in infants and childeren suggest a benefit in the improvement of hematologic iron markers but iron supplementation may not significantly improve growth and neuromotor development [21–24]. It is important to consider the effects of iron rich food on iron status and growth, along with the possible risk of infections, particularly in developing countries where water supplies may not be safe [25]. Our findings regarding growth are in line with that of Kramer and Kakuma, who found non-significant differences in linear growth in infants introduced to solids before 4 months and those breastfed until 6 months, and on which the WHO recommendation was largely based [12]. We identified only one observational study that opposed the findings of Kramer and Kakuma. It assessed the effect of introducing CF at exactly 4 months of age versus 6 months. This finding is due to our stricter criteria, as these are the controversial time points that most of the organizations’ recommendations fit in. A previous systematic review identified significant growth improvements with provision of solid foods [26, 27], but this review included studies conducted on moderately malnourished infants, where the ones included in our review were all healthy.

More evidence is needed to agree on the optimal timing of introduction of solids to exclusively breastfed infants. In future studies, ideally multi-center ones with long-term follow up, special attention should be given to hematological results to achieve a definitive conclusion on this important issue.

### Limitations

The included studies had short follow-up periods in which to measure the impact of complementary food introduction. Longer term outcomes remain uncertain. Another limitation of our review is the inclusion of studies with small sample sizes. Finally, pooled data analyses could not be performed for all the outcomes due to the differences in the outcome measures assessed in the individual studies.

## Conclusion

Encouraging exclusive breastfeeding is a desirable goal for health care professionals as there is consistent evidence to support breastfeeding. However, the generalized recommendation to introduce solid foods at 6 months of age may not be optimum for all healthy, breastfed infants. Based on the findings of this review, the iron status of healthy full-term infants could be positively altered by an earlier introduction of complementary foods, leading to preservation of infant iron stores. Furthermore, there may be value in changing the current statement regarding solid introduction from a fixed time (6 months) to a range of time (4–6 months), leaving individual decisions to health care professionals and parents. Larger randomized controlled multi-center trials in developed and developing countries are needed to further investigate the differences in outcomes after introduction of solids before and at 6 months of age.

## Additional file

Additional file 1:
**PRISMA 2009 Checklist.** (DOC 62 kb)

## Abbreviations

CF: Complemenatry food
MD: Mean difference
RCT: Randomized controlled trial
